# A Security Concept Based on Scaler Distribution of a Novel Intrusion Detection Device for Wireless Sensor Networks in a Smart Environment

**DOI:** 10.3390/s20174717

**Published:** 2020-08-21

**Authors:** Kenneth Rodolphe Chabi Boni, Lizhong Xu, Zhe Chen, Thelma Dede Baddoo

**Affiliations:** 1 College of Computer and Information Engineering, Hohai University, Nanjing 211100, China; boni_kenneth@yahoo.fr (K.R.C.B.); chenzhe@hhu.edu.cn (Z.C.); 2 College of Hydrology and Water Resources, Hohai University, Nanjing 210098, China; mzdede2010@gmail.com; 3 Binjiang College, Nanjing University of Information Science & Technology, No. 333 Xishan Road, Wuxi 214105, China

**Keywords:** smart environment, IDS, feedback signal, trust table concept, sensor gate, security hole

## Abstract

Following the significant improvement of technology in terms of data collection and treatment during the last decades, the notion of a smart environment has widely taken an important pedestal in the science industry. Built in order to better manage assets, smart environments provide a livable environment for users or citizens through the deployment of sensors responsible for data collection. Much research has been done to provide security to the involved data, which are extremely sensitive. However, due to the small size and the memory constraint of the sensors, many of these works are difficult to implement. In this paper, a different concept for wireless sensor security in smart environments is presented. The proposed security system, which is based on the scaler distribution of a novel electronic device, the intrusion detection system (IDS), reduces the computational functions of the sensors and therefore maximizes their efficiency. The IDS also introduces the concept of the feedback signal and “trust table” used to trigger the detection and isolation mechanism in case of attacks. Generally, it ensures the whole network security through cooperation with other IDSs and, therefore, eliminates the problem of security holes that may occur while adopting such a security technique.

## 1. Introduction

Scientific expertise today seems to be greatly focused on the security of wireless sensor networks (WSNs) and their performance. A WSN is a mobile ad-hoc network that is exposed to many threats. From the improvement of the technical system embedded in the sensors to the development of security techniques to offset the numerous attacks that these networks can face, nothing is left behind in the research into wireless sensor networks.

The various research carried out at great cost by researchers, institutions, academies, and even universities, confirms this impression: security performance has become the subject of the deployment of scientific ingenuity. Confidentiality of the data collected by the sensors, availability, integrity, or even intrusion detection, are the various essential security expectations to be met. To meet these requirements, several experts place particular emphasis on the use of encryption techniques for data protection (see works proposed by Zhang et al. [1], Gover et al. [2], Krontiris [3], Kumar et al. [4], Perrig et al. [5], Wang et al. [6], Zhu et al. [7], Girao et al. [8] and also Raj et al. [9]). These techniques are generally based on the utilization of keys for data encryption and decryption. Other experts going further have opted for the development of certain algorithms used by these sensors to identify malicious behavior within networks. Studies were done by Patil et al. [10], Singh et al. [11], Sonu et al. [12] and Jamshidi et al. [13], who presented techniques that address specific attacks like DoS (denial of service) attack, wormhole attack, sinkhole attack, and Sybil attack, respectively. These encryption techniques and algorithms suggested by the previous studies are implemented into the sensors themselves.

With the rise of the Internet of things (IoT) notion and the need to have access to collected data in a smart way, the concept of a cloud platform has also been rapidly gaining popularity in smart environments. The feature of a cloud platform is that any user can have access to the information in real time or later on. In order to prevent from data leaking during the communication, Wazid et al. [14] have presented a new lightweight authentication mechanism in cloud-based IoT environments. The mechanism suggests a joint authentication between the user and the sensor. The joint authentication allows the two devices to create a session key which will be used for any other communication. Also called LAM-CIoT (lightweight authentication mechanism in cloud-based IoT environment), the peculiarity of the proposed security relies on the fact that any user can have access to the data from the cloud server where the data are stored or directly from the sensor. The security part is performed with the help of a trust authority which is in charge of the network components registration.

This idea is also presented by Challa et al. [15], who introduced a novel authenticated key agreement scheme for cloud-assisted environments, especially for smart grid environment. Based on a cloud-assisted Cyber-Physical-System, the mechanism ensures two authentication levels (between a user and a cloud server, between a smart meter and a cloud server, etc.).

To sum up, if these presented mechanisms can help in securing the connection between a user and a sensor, or a sensor and the cloud (with the utilization of deterministic key management schemes), a problem concerning the sensor energy efficiency still remains. In fact, the mechanisms suggest the creation of a session key based on computational skills, which requires more energy. Also, by maintaining the created session key at the sensor side for future communication with a specific user, the problem of memory availability will soon occur since in this particular network configuration, the sensor may communicate with several users. Therefore, the implementation of the techniques and methods proposed by these authors is hard to achieve and may affect the performance of the wireless sensor networks since the sensors are really tiny and do not enjoy a large memory size.

Furthermore, Saia et al. [16] reported on the use of a blockchain-based distributed paradigm for data exchange between wireless-based devices, which they defined as Internet of Entities (IoE). The authors believed that this form of communication based on the decentralization model and a certification function offered by data exchange paradigms such as cryptocurrency could be useful in applications in Security, eHealth, and Smart Cities, where the trackers, which are the wireless devices, and in this case sensors, connect with entities which are people or things through a blockchain ledger to ensure the anonymity of communication and therefore, preserve data availability and freshness. However, data confidentiality, integrity, and authenticity could be compromised when this blockchain ledger becomes compromised.

Some other studies have suggested the use of intrusion detection systems (IDS), a security scheme which aims at detecting anomalies and attacks (actions that seek to jeopardize the system security factors) in wireless sensor networks. For example, Alrajeh et al. [17] in a review of intrusion detection systems for wireless sensor networks reported that generally, the IDS can only detect attacks but cannot prevent or respond; instead, it alerts network administrators about possible attacks well in time to stop or reduce the impact of the attack. This solely alerting mechanism of most IDS schemes as discussed by the authors could become problematic in cases where the sensors are deployed in hard to reach areas and the kind of threat requires the network administrator to physically check out the system for threat isolation.

Reviews of different intrusion detection schemes based on machine learning reported in the literature by da Costa et al. [18] reveal that the attacks on wireless sensor networks and IoT devices keep increasing and evolving, making security in these aspects a challenge. However, computer scientists worldwide are also progressing in the detection of these intrusion attacks and formulating various mechanisms (intrusion detection techniques) to identify them, although most of these mechanisms are specific-attack-based and usually involve time-, energy-, and memory-consuming processes in their intrusion detection processes.

Furthermore, Godala and Vaddella [19] in a recent study reported that IDS techinques in current literature comprised of: signature-based IDS, anomaly-based IDS, specification-based IDS, and Hhybrid-based IDS. Whereas the signature-based IDS mechanisms are used for known threats, the anomaly-based IDS techniques aim to identify unknown attacks by employing statistical, data mining, machine learning, and artificial intelligence approaches, among others, while the specification-based IDS schemes detect both known and unknown attacks and the hybrid-based IDS methods apply several combinations of the other techniques for intrusion detection. Their study revealed that the majority of the wireless security mechanisms based on the various IDS approaches focus only on specific types of attacks centered on specific layers of the WSN, without much consideration on other layer attacks, and also need more time and memory for implementation because of the techniques adopted for these intrusion detection schemes.

Considering these facts, this study, therefore, introduces a novel intrusion detection system (IDS), which is a device capable of ensuring a perfect security scheme for wireless sensor networks inside a smart environment. The device, which is also a sensor with much more energy power and memory space, assumes the computational functions meant for the sensors in the network and embraces the security aspect of the network by filtering all incoming and outgoing communication. Embedded with a sniffing program, the IDS makes use of a “trust table” which is created during the initialization of the wireless sensor network to maintain a certain trust level in the deployed environment by separating the genuine and malicious sensors, and therefore equips the intrusion detection device (IDS) with the ability to analyze all the sensor network traffic patterns. The advantage of such a device is that, regardless of the nature of the attack, the security is still guaranteed since the device makes use of its previously saved sensor data to estimate whether or not the new captured events in the network are genuine. We also suggest the deployment of several of the proposed IDSs (scaler distribution) working jointly to ensure the entire network security of the smart environment. That is, the IDS participates in the transmission while the data is being sent from one sensor area to another by making use of some cryptographic encryption techniques. This also allows the IDS to eliminate the dead zones, referred to as security holes, in the smart environment.

### Motivation and Novelty

The proliferation of wireless sensor networks (WSNs) in human livelihood is expected to increase due to the active developments in technology and widespread application of the Internet of Things paradigm in computer networks across the world [18]. Attacks on WSNs are inevitable due to this interconnectivity and therefore, network security has become a present-day challenge. However, destruction and loss of information can be prevented and/or greatly minimized with the appropriate wireless sensor network security scheme.

Godala and Vaddella [19], after reviewing several different current IDS security techniques, recommended the development of a cross-layer IDS that can detect the different attacks, of which may occur in different layers of the WSN. This is the innovation which this study brings across, where the IDS proposed in this study performed a hybrid-based intrusion detection system on all layers because it is a novel device equipped with sufficient memory, energy, and security to perform attack detection and isolation simultaneously on sensors, as well as the data being processed and transmitted.

Neisse et al. [20] also offered a model for intrusion detection based on a system of trust relationships to govern the communications among devices in IoT-based environments. In our study, we go further in securing this trust relationship through a sensor trust table in the IDS device to prevent the sensors from having to save their multiple interconnection relationships in their already insufficient memory space.

Finally, to the best of our knowledge, no study has suggested the use of an intrusion detection system which does not involve computation by the wireless sensors themselves in the detection process. The studies reviewed depend on the sensors actually performing the attack detection work before forwarding to the next level in the network or the base station.

Also, the reviewed works do not involve automatic isolation of threats in the wireless network but depend on the isolation from the base station when threats or attacks which disrupt the network functions are detected, which in cases of eavesdropping, the attack message might not get to the base station on time for timely attack isolation, causing much more loss of information in the wireless sensor network deployment area. Our work involves automatic isolation of threats before further investigation by the base station administrator, for better data protection.

## 2. Related Work

### 2.1. Existing Relation between Sensors and Smart Environments

**Figure 1:**
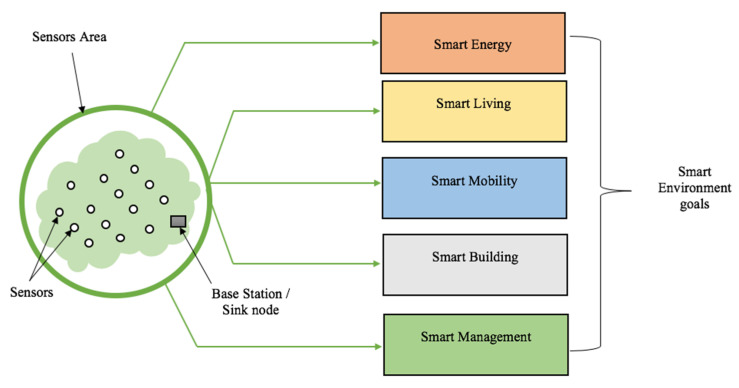
Overview of a smart environment and its goals.

Built on information networks around citizens, a smart environment is a “hi-area” composed of several electronic sensor devices that collect some specific data from a specific geographic area (sensor area). These data are processed and analyzed to be made available to the users. Designed to optimize resources and for sustainable development, the technology behind the smart environment is to provide a livable environment. From smart mobility, smart building, smart infrastructure, smart energy, and smart management, smart environments have been contributing immensely in the analog to digital transition of humankind. The basic components in a smart environment are depicted in Figure 1.

The collected data are sent to a base station (BS)/unit control or sink node via a particular communication channel. On arrival at the base station, the data are processed for further use or a smart purpose. A sensor is a device with the ability of physical condition or quantity measurements such as temperature, pressure, moisture, and concentration measurement, leading to the generation of a suitable measurable response in the form of output signals [9]. Sensors can be classified based on these criteria [21]: primary input quantity (measured), transduction principles (using physical and chemical effects), material and technology, property, and applications.

The role of a sensor relies on sensing, data processing, and communicating [22], especially in a smart area. Thus, a standard sensor node consists of assorted components: a radio transceiver with an antenna with the ability to send or receive packets (data), batteries delivering the required energy supply, and a microcontroller which processes the data and arranges the different related tasks between the sensor and the various other sensors sensing the environment data, because each node or sensor can communicate with all other sensors wirelessly. By communicating with one another, the sensors form a particular network, which is called the wireless sensor network (WSN).

A wireless sensor network (WSN) is a network consisting of several wireless devices (nodes or sensors) deployed randomly over a given geographical area and powered by batteries. The different topologies that can be adopted in wireless sensor networks are: star network, mesh network, and hybrid star network [23].

### 2.2. Wireless Sensor Networks’ Vulnerabilities and Threats

Wireless sensor networks are constantly exposed to severe attacks due to some weaknesses and, most importantly, due to the involved data. A system is vulnerable when there is a weakness in the design or the execution of an information system. This weakness could be deliberately or involuntarily exploited to affect the confidentiality, integrity, or availability of data [24]. Despite the many advantages that they provide in terms of data sensing and monitoring, WSNs are still vulnerable to threats. Energy constraints, memory limitation, unreliable communication, higher latency in communication, unattended operation of the network, deployment in an environment more open to attacks, close interaction with the physical environment and with people, transmission range, fault tolerance, self-organization, and scalability are just a few of these vulnerabilities [25,26].

Due to the high importance (sensitivity) of the collected data, WSNs are mostly confronted with all kinds of attacks, including physical attacks (interaction with people due to the environment), where anyone, malicious or not, can have physical contact with a sensor of the network, thereby disrupting its proper functioning. These attacks against WSNs are of different types and are categorized as invasive or non-invasive, and usually comprise side-channel attacks such as power-, timing-, or frequency-based attacks [27]. The WSN attacks can also be classified as passive or active, internal or external, different protocol layer attacks, stealthy or non-stealthy, cryptographic, or non-cryptographic attacks [28].

**Figure 2:**
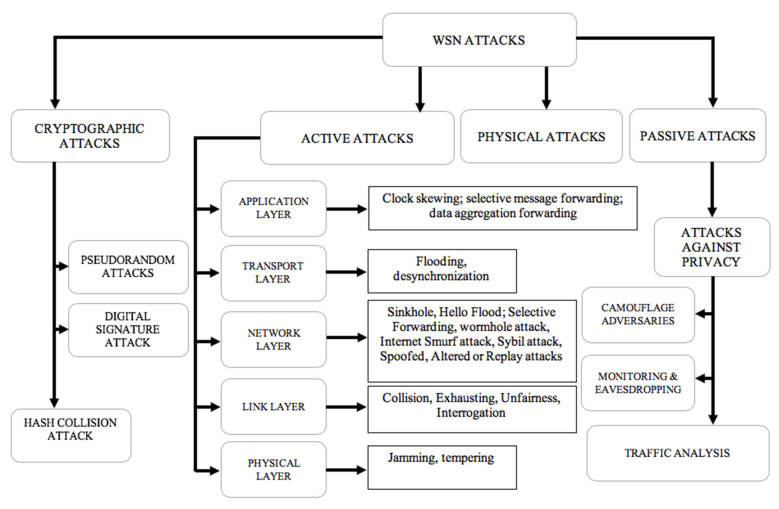
Wireless sensor network (WSN) attacks classification.

Figure 2 gives a classification of the attacks that can be performed against the WSNs. A physical attack is mentioned when there is physical contact of the sensor with the physical environment, such as individuals intentionally or unintentionally disrupting the network through touch or movement and thus destroying the network. With inactive attacks, a malicious person (hacker) attempts to manipulate the transmitted message. The manipulation concerns the modification or the removal of the message that is being sent in the network. By doing so, the hacker can inject his traffic or replay messages that have already been sent. This technique causes a disturbance of the network operation or a denial of service (DoS) [29]. The active attacks can be initiated on five different layers of communication protocols [28,30]: physical layer attacks (where an attacker easily jams or intercepts any ongoing signal) [31], link-layer attacks (here, the attack is performed against the Media Access Control Protocol [32]), network layer attacks (including hello flood, wormhole attack, Sybil attack, selective forwarding, spoofed, and altered or replayed routing information [33,34]), transport layer attack (which mainly concerns flooding and desynchronization [33]), and the application layer attack, where an attacker attempts to overload the network nodes with sensor stimuli, triggering the network to forward massive volumes of traffic to a base station [35]. The application layer attacks include clock skewing, selective message forwarding, and data aggregation forwarding [36].

The passive attack is the kind of attack which occurs when there is unauthorized monitoring and listening of the communication channel to obtain the data exchanged in the network without disrupting the communication [37]. An example includes camouflage adversaries, monitoring, eavesdropping, and traffic analysis.

Other WSN attacks which are cryptographic-based are performed against some crypto functions of the network. Examples include digital signature attack (where an attacker performs the attack based on three methods: using a list of messages that were previously signed by the targeted sensor, using a specific message that it wants the victim to sign, and by using the verification algorithm), hash function attack (where the attacker reproduces a valid certificate matching to the hash collision), and pseudo numbers attack (to compromise the message freshness by disclosing the cryptographic key) [28].

Considering the impact that these attacks could have on a wireless sensor network, some works have been presented by several researchers. For instance, Patil et al. [10] suggested a technique based on the cooperative fuzzy artificial system (Co-FAIS), which addresses the issue of denial of service (DoS) attack. Singh et al. [11] also recommended a wormhole-resistant hybrid technique based on two mechanisms (watchdog and Delphi) to tackle the problem of a wormhole attack. Furthermore, Sonu et al. [12] presented a methodology based on a unique identifier and a secret key assigned to each sensor of the network to detect the Sybil attack. In addition, Jamshidi et al. [13] introduced an algorithm that allows the sensors to monitor and decide when facing a Sybil attack. A class of machine learning algorithms based on support vector machines (SVMs) was proposed by Kaplantzis et al. [38] to approach the selective forwarding attack problem. These proposed methods or algorithms, in general, are designed to solve a particular attack. However, they present a challenge of inefficiency to the security of the wireless sensor network under the multiple attacks that may be performed against a sensor network at a time. Therefore, it is not advisable to deploy a security scheme based on a specific attack detection system. This is to say that, considering that all these previously suggested WSN security schemes are specific-attack-based, it will be impossible to detect and eliminate a Sybil attack when the wormhole-resistant hybrid technique by Singh et al. [11] is incorporated into the wireless network system. This security constraint is observed among all the existing works. Therefore, they do not provide a comprehensive security scheme to the wireless sensor network and the smart environment as a whole, in cases of multiple attacks in this age of technological advancement, where malicious individuals have developed several attack techniques.

The scientific environment continues to upgrade in the search for better IDS schemes to detect the various attacks WSNs are prone to.

Otoum et al. [39] developed a hybrid IDS framework, which they named Adaptively Supervised and Clustered Hybrid IDS (ASCH-IDS), for wireless sensor clusters to monitor the sensory data aggregation stage of critical infrastructures, such as power grids or residential microgrids, which continually supervises the network for misuse detection or anomaly detection. Their system is based only on the data being collected and not on the sensors collecting such data. However, in some attack cases in wireless sensor networks, such as eavesdropping, where the attacker does not disrupt the system functions or the data but spies on the information being processed and transmitted in the network to collect these sensitive data, is not considered by the authors.

Furthermore, Zhang and Xiao [40] developed an intrusion detection scheme using a negative selection algorithm based on spatial partition in hierarchical wireless sensor networks. They reported that this algorithm is an upgraded version of the same algorithm used previously in the literature but with amelioration in the time efficiency, energy consumption, and sensor node resources’ preservation, which is mostly not considered in the other existing literature of IDS mechanisms. However, the memory space of the sensors as these algorithms are implemented in them was not fully considered.

Additionally, Han et al. [41], in their study to improve the network lifetime by reducing high energy consumption due to previous IDS mechanisms, also designed an intrusion detection model based on game theory and an improved autoregressive theory model. They showed that this new model takes the energy consumption of the intrusion detection process into consideration by being time-efficient, since in wireless sensor networks, the running time of the network processes is the main indicator of energy consumption of the system, also without consideration for sensor memory space.

It should be noted that the recommended IDS schemes in the current literature, including the ones discussed earlier involving data mining [42,43,44], machine learning [39,45], some form of artificial intelligence [46,47], or the like, are implemented in the sensors themselves, with the majority of them being specific-attack-based. Also, most of the authors comment on the small size of sensor batteries and memory space, but without much focus on the amelioration of the effects of the implemented security schemes on the efficiency and shelf life of the sensors involved, especially in terms of sensor memory space.

Therefore, given the fact that sensors are not energy-efficient due to their small size, allowing the sensors to participate in the intrusion detection process by uploading some algorithms or methods locally inside the sensors themselves is deemed inadequate and unsatisfactory, as presented by Krontiris [3]. This is also the case of the cryptographic techniques that require the sensors to compute some functions based on key management systems, as proposed by Zhang et al. [1], Gover et al. [2], Krontiris [3], Kumar et al. [4], Perrig et al. [5], Wang et al. [6], Zhu et al. [7], Girao et al. [8], and Raj et al. [9], among others. Since the main function of the wireless sensors in a WSN or smart environment is communication, sufficient energy and a stable source of energy in or for these sensors are required for their smooth performance. The further the receiver sensor is from the transmitter sensor, the more the energy consumption will augment.

Assuming *E_c_* to be the energy consumed while transmitting a message, *M*, over a distance, *d*, the value of the consumed energy is obtained by:

(1) $$\;E_{C}=E_{Tx}\;M+E_{am}\;d$$

where *E_Tx_* is the transmission energy and *E_am_* is the transmit amplifier energy. In this case, whenever the message increases in size, and the distance increase in length, the energy consumed by the transmitting node will also increase. Another important factor affecting the sensor energy consumption is the time. Consequently, the energy consumed while transmitting the message over a period of time (*t*) is obtained as follows:

(2) $$\;E_{C}=E_{Tx}\;M.t\;+E_{am}\;d.\;t$$

(3) $$\;E_{C}=t(E_{Tx}\;M+E_{am}\;d)$$

Taking *E_F_* as the energy needed to compute some functions either based on cryptographic techniques of key management systems or algorithms which are specific-attack-based, the energy consumption is now obtained by:

(4) $$\;E_{C}=t(E_{Tx}\;M+E_{am}\;d)+E_{F}$$

It is, therefore, necessary to introduce another approach for WSNs, especially in a smart environment. This new approach should be able to reduce the computational function of the sensors and protect the WSNs as well.

## 3. The Newly Proposed Intrusion Detection Device (IDS)

### 3.1. The IDS Model 

**Figure 3:**
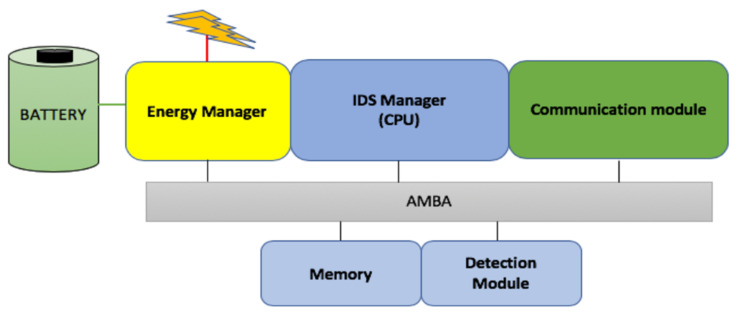
The intrusion detection system (IDS) architecture overview.

The new proposed intrusion detection system (IDS) is an electronic device capable of ensuring a perfect security scheme for wireless sensor networks inside a smart environment. The IDS architecture is centered on three (3) functional modules: an energy module, a detection module, and a communication module. The device supports the IEEE (Institute of Electrical and Electronics Engineers) 802.15.4 protocol and is also equipped with a Random-Access-Memory (RAM) and a Read-Only-Memory (ROM) for data and programs’ storage. The modules are interconnected using an advanced microcontroller bus architecture (AMBA). The AMBA is a commonly used standard to interconnect the System on Chip (SoC) design [48]. A general overview of the IDS is presented in Figure 3.

The energy module empowers the device with sufficient energy, while the detection module ensures the network monitoring and the execution of the detection and the isolation process. On the other hand, the communication module is responsible for wireless communication functions. It broadcasts all the necessary instructions, especially during the transmission of the feedback signal. The communication module also includes a MAC (Medium Access Control) and physical layer.

To guarantee an efficient power system to the module, the battery is empowered by a solar power system. The solar power system will allow the system to be functional at all times and enjoy a longer life period. This will be useful in cases where the device has to be deployed in a hostile environment where human access would be difficult.

The major role of the IDS is to take over all the computational functions of the sensors since the sensor energy resource and memory size is a big challenge. It is embedded with a sniffer program to analyze the entire sensor network environment activities and report all malicious behavior in the network. From route requesting, route establishment, message transmission, and message reception to network configuration, the device captures, without modifying, all the flowing data over the sensor network. This is done based on the watchdog approach that allows the device to overhear all ongoing communication inside the network. The IDS works according to all the network protocols deployed in the sensor network. All the sniffed information is processed and stored in the device. The sniffer program works along with other programs which compare the sniffed data to the one that was previously stored in the IDS memory. Based on the comparison results, a decision is made by the IDS (whether the network is secure or not).

The IDS introduces the concept of the “trust table” (TT), which is composed of two tables: the genuine sensor table (GST) and the blacklisted sensor table (BST). Each table is composed of node identification, geographic location, and, if possible, the serial number of the sensor. The GST is important as it allows the IDS to confirm whether or not a sensor can be treated as a threat. The TT is a database made up of: the number of sensors (NS), displaying the total number of sensors in a sensor area, the number of backlisted sensors (NBS), and the ID, which is the device identification in the network. The TT, like the other programs, is created or installed during the sensors network initialization or deployment.

### 3.2. The IDS Performance against Attacks 

Providing a good security scheme for the WSNs should be the priority of network administrators, especially since different hacking techniques are being developed and used by malicious people to retrieve sensitive data. Our proposed new intrusion detection device has superior authority over all sensors and data inside the network.

**Figure 4:**
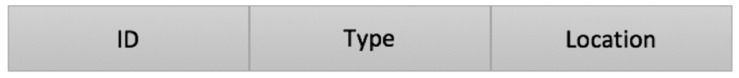
Malicious sensor information sent in the feedback signal (F.S).

Another important role of the IDS is to broadcast the feedback signal (F.S). The feedback signal is a broadcast message which is sent to all the genuine sensors in the network to activate threat isolation in cases of attacks. The feedback signal is an alert that is sent to the sensors with specific instruction. The packet frame mainly contains data which includes necessary information about the threat. Figure 4 presents some of the information that is in the F.S.

**Figure 5:**
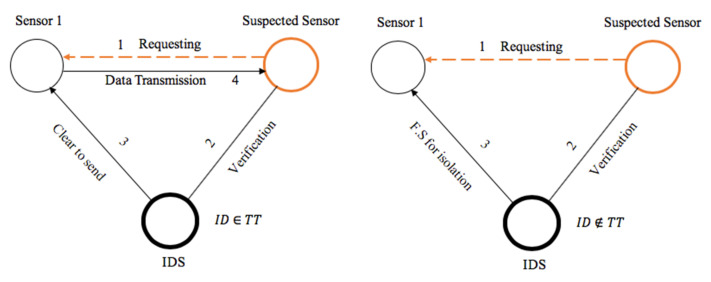
Proposed scaler distribution of IDSs in a smart environment.

In computer networks, each device is set to have unique identification (ID). This ID is used to identify the device inside any network. The intrusion detection system, IDS, after detecting the intruder or the sensor acting maliciously, includes its ID into the F.S. The location refers to the position of the malicious sensor inside the network. All the other sensors avoid this position while a new route or path is being calculated for data redirecting. The type refers to the nature of the attack or threat. The role of the IDS in a sensor network during transmission is given in Figure 5. 

As presented in Figure 5, every sensor requesting for a route or data transmission is considered suspect unless the IDS provides formal approval about its identity and “innocence” inside the network. The efficiency against this kind of attack is proven, especially when an attacker creates an illusion to the sensor transmitting the data that the selected path is the shortest path for data transmission to the sink node. The detection and isolation mechanism presented in Figure 5 is implemented as follows:
START S_1_: Sensor_1 SS: Suspected_Sensor SS_id_: Suspected_Sensor_ID TT: Trust_Table BL: Blacklisted_List FS: Feedback_Signal_for_isolation r: SS_request CTS: Clear_to_Send_message S: All_Sensors_in_the_network   If SS sends r to S_1_ then // Intruder asks sensor_1 to share information with it  Get SS_id_; // The IDS acknowledges the suspected sensors identity   If SS_id_ ∈ TT then // IDS determining if the suspected node is in the trust table  Deliver CTS to S_1_; // IDS delivering a clear to send signal to sensor_1  Else  Deliver FS to S; // IDS delivering feedback signal to all the sensors, including Sensor_1  Store SS_id_ in BL; // IDS storing the suspected sensors ID in the backlisted list  End if  End if End if END

Our proposed security system is also able to detect and isolate any previous genuine sensor that later turned rogue inside the network. The cause of a sensor turning rogue could be due to the occurrence of some internal failure. One of the well-known behaviors of such a threat is that the involved sensor could start dropping the received packets. This could have a severe impact on the sensor network as it leads to data loss or energy drainage for both the transmitting node and the rogue sensor. In this kind of situation, the involved detection and isolation steps when packets are being dropped in the network are presented below:
START S_1_: Sensor_1 RS: Rogue_Sensor RS_id_: Rogue_Sensor_ID TT: Trust_Table P: Packets BL: Blacklisted_List FS: Feedback_Signal_for_isolation r: SS_request S: All_Sensors_in_the_network   If RS drops P then // rogue sensor starts dropping packets sent by Sensor_1  Alert S_1_; // alert sensor_1 to search for new route  Deliver FS to S; // IDS delivering feedback signal to all the sensors, including Sensor_1  Store RS_id_ in BL; // IDS storing the suspected sensors ID in the backlisted list  End if END

Every IDS periodically conducts a check-up of the sensor network. This enables the IDS to ensure the accuracy of previously stored information to make sure nothing has been changed, just like a sensor updating process. The IDS starts by getting access to the “trust table.” Basically, the system verifies if the number of the previously stored sensors, *NS*, is still the same compared to the new updated number. This is done by just comparing two values. Considering *NS*, the value of the stored number of sensors, and *NS*’, the updated value of the stored number of sensors at the present time, *Ta*. If the values are the same (*NS* = *NS*’*_Ta_*) then the IDS concludes that there were no new malicious entries. On the other hand, if *NS* ≠ *NS*’, then the *IDS* concludes that there have been sensor entries into the network. Therefore, the intruder is located, and its information is saved in the device memory in the list of blacklisted sensors and broadcasted to the other sensors of the network through the feedback signal (F.S). The sensors, upon receiving the message, proceed to the isolation of the blacklisted sensor by avoiding communication with it in the future. If *D* is the intrusion detection process of an *IDS*, the detection process can be presented as:

(5) $$D=\{\begin{array}{c} safe\;if\;NS_{\;}=N{S^{\prime }}_{Ta}\\ unsafe\;if\;NS_{\;}\neq N{S^{\prime }}_{Ta}\; \end{array}$$

The steps of the algorithm are presented below:
START i: int k: int n: int SA: Sensor_Area NS’: Number_of_Sensor_at_time _a NS: Number_of_Sensor_in the_network_initially TT: Trust_Table IS: Intruder_Sensor IS_id_: Intruder_sensor_ID BL: Blacklisted_List FS: Feedback_Signal_for_isolation S: All_Sensors_in_the_network  k = 0 For i → 0 to n i = i + 1 k = k + 1  If k = NS then // IDS comparing the number of sensors NS’ in the sensor area at present to   the one in TT  Conclude SA is safe;  Else  Locate IS;  Get IS_id_;  Broadcast F.S to S; // IDS Broadcasting an F.S which contains the intruder sensors ID and   location  Store IS_id_ in BL; // IDS storing the Intruder_Sensor ID in the backlisted list  End if End if END

### 3.3. Scaler Distribution of the IDS and the Cooperation

**Figure 6:**
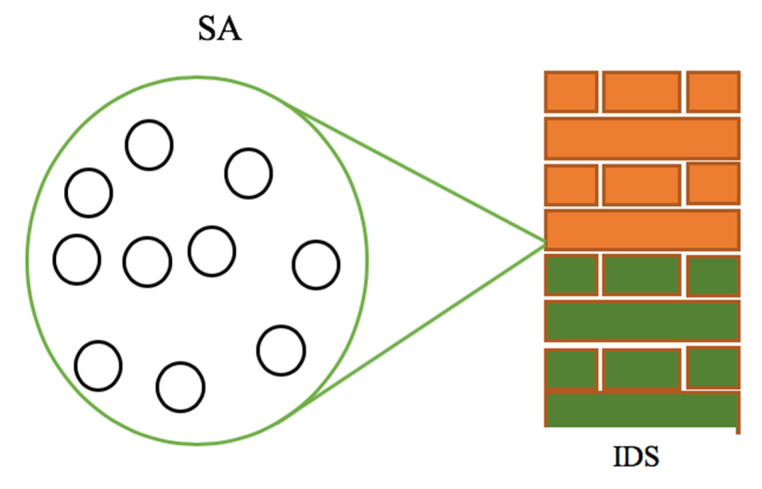
The intrusion detection device building a virtual fence around the sensor area.

To increase its performance efficiency, several sensors are allocated to a particular IDS. By doing so, the IDS can build a virtual fence around the sensors under its authority, and all incoming and outgoing transmissions are filtered. An overview of the IDS building a virtual fence around the sensor area is presented in Figure 6.

Figure 6 shows a sensor area (SA) composed of ten (10) sensors. The IDS monitors these sensors. To be able to manage a given number of sensors, the IDS is set to have a communication module capable of covering a wide communication range. This enables the IDS to ensure its monitoring functions and to protect the network from attacks.

A smart environment may contain thousands of sensors. We therefore propose the implementation and use of several distributed IDSs in the smart environment. The IDSs are to cooperate in a perfect symphony. This cooperation will still guarantee the security of the data while being transmitted from cell to cell.

**Figure 7:**
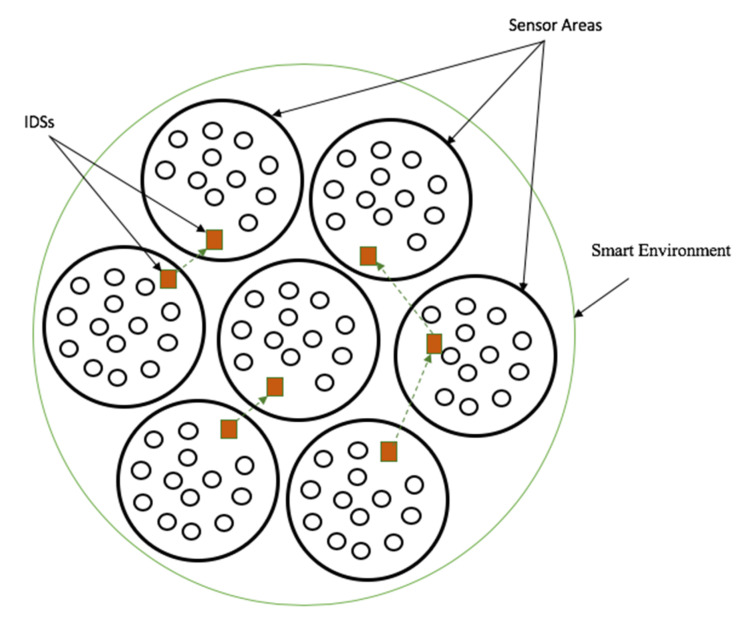
Proposed scaler distribution of IDSs in a smart environment.

Figure 7 shows a smart environment that includes several sensors distributed into various sensor areas. Also called cells, a specific IDS manages each sensor area. The IDSs are also involved in inter-cells data transmission, where they ensure the authenticity of the incoming and outgoing data from their respective cells to the other cells in the smart environment. 

**Figure 8:**
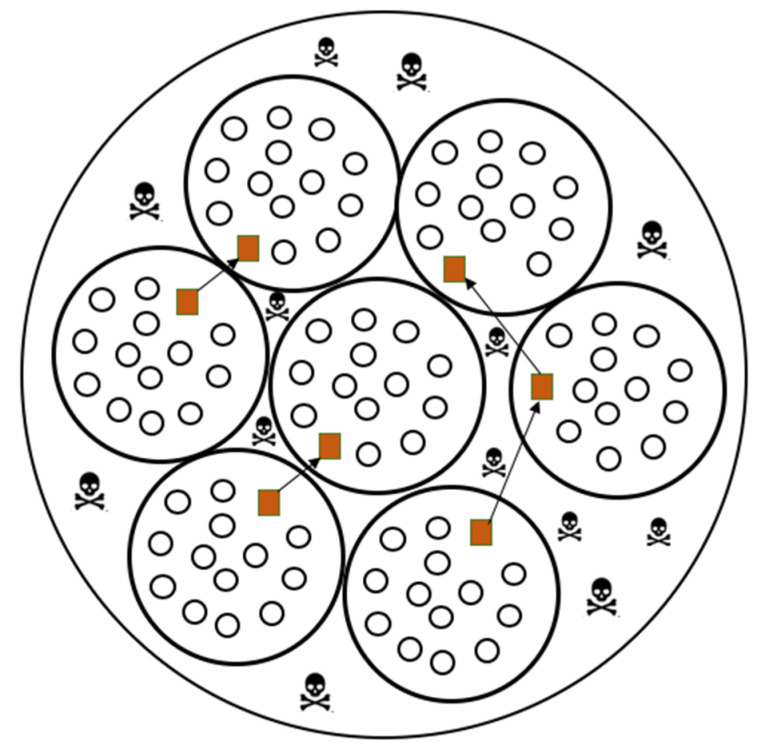
Dead zones in the distribution of IDSs in a smart environment.

Between each IDS deployed sensor area exists some unattended to gaps, referred to as dead zones in this study. Figure 8 gives an illustration of the dead zones.

As presented in Figure 8, the dead zones are the ones that are not covered by any *IDS*. The presence of the dead zones might be a vulnerability to the network, thus leading to a security hole. A malicious person can also try to intercept or alter the data while it is passing through this hole, thereby disrupting the security of the entire smart environment. To guarantee the security of transmitted data while it is in a dead zone, each IDS encrypts the data before forwarding from its sensor area to another sensor area.

Therefore, the first step of the entire proposed security system occurs at the beginning of the deployment of the network. In fact, during the initialization, each IDS receives many sensors under its authority. The trust table is then created, and all the sensors’ information is saved inside the IDS. The IDSs also acknowledge the sensor gate identity. The sensor gate (the nearest sensor to the *IDS*) is the last sensor in the data routing process. During the initialization, each IDS is also loaded with some keys based on the cryptography techniques. The IDSs are loaded with essential elements that will be used during their entire existence. An example of an *IDS*s key elements for *IDS*1 is illustrated by:

(6) $$IDS1=\left\{\left(Pr_{1},\;Pk_{2},\;\ldots ,\;Pk_{n}\right)\right\}$$

where *Pr*_1_ is the *IDS*1 private key used for data decryption, *Pk*_2_ is the *IDS*2 public key used for data encryption, and *Pk_n_* is used by *IDS*1 to encrypt data sent to *IDS*n.

The keys are generated based on mathematical principles by randomly selecting two prime numbers p and q, and an odd number e, which is coprime to φ (n), such that [49]:

(7) 1<e<φ(n)

**Figure 9:**
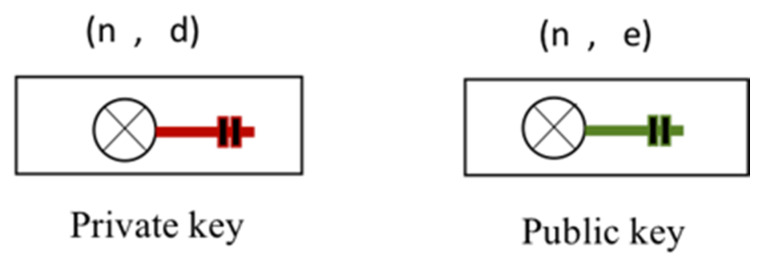
Public and private key mathematical definition.

After the number n=p.q the number $d=e^{-1}mod\left(\varphi \left(n\right)\right)$ is also computed using Euclid’s algorithm. In this case, (n, e) is the public key while (n, d) is the private key. An illustration of the private and public key definitions is given in Figure 9.

After the generation of the keys, the private one is kept secret by the transmitting IDS while the public one is broadcasted into the network.

Considering a message, *M*, to be transmitted to a user, *A*, such that: $\;0\leq M\leq n_{A}$, the encrypted message also called ciphertext, *C*, is obtained through:

(8) $$C=E_{A\left(M\right)}=mod\;n_{A\;}M^{eA}$$

where E represents the encryption algorithm and (*n_A_*, *eA*) is the user, *A*, public key.

**Figure 10:**
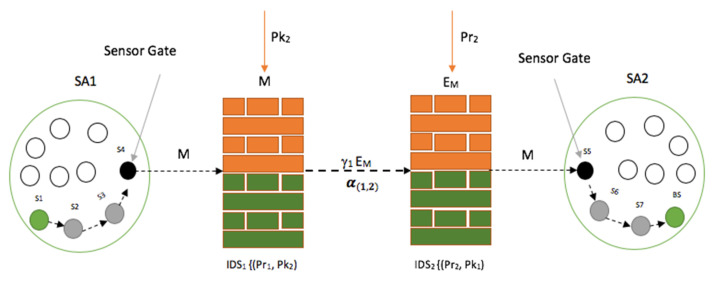
A cooperation mechanism between two IDSs.

Figure 10 demonstrates the cooperation between two (2) IDSs, IDS1 and IDS2, in the scaler distribution process of IDS deployment in a smart environment to ensure the perfect security scheme, where *M* is the transmitted data, SA1 is sensor area 1, SA2 is sensor area 2, *Pr*_1_ and *Pk*_1_ represent *IDS*1 private and public keys respectively, *Pr*_2_ and *Pk*_2_ are *IDS*2 private and public keys respectively, α(1,2) is the cooperation mechanism between *IDS*1 and *IDS*2 in providing a perfect security scheme during the data transmission, and γ_1_ is the transmission channel used by *IDS*1 to transmit the encrypted message, *E_M_*.

Considering the demonstration in Figure 10, we assume that sensor 1, which is located in sensor area 1 (SA1), is transmitting data to the sink node (base station) located in sensor area 2 (SA2). Both of the IDSs participate in the intrusion detection process and ensure the success of the message forwarding until it reaches the sink node or base station.

The general equation of the cooperation mechanism between IDS1 and IDS2 and with *n* number of IDSs can be demonstrated as:

(9) $$\alpha_{\left(1,2\right)}=\{\begin{array}{c} Y\;if\;\left(IDS_{\left(1,2\right)}E_{M}\cdot \gamma \right)\;is\;TRUE\\ N\;if\;\left(IDS_{\left(1,2\right)}E_{M}\cdot \gamma \right)\;is\;FALSE \end{array}$$

where *γ* is the transfer channel between IDS1 and IDS2. Therefore, if IDS2 is able to decrypt the data sent by IDS1, then the cooperation is successful, otherwise, the cooperation has failed, and the data is not transmitted.

As shown in Figure 10, IDS1 and IDS2 are responsible (locally) for the message transmission and the detection of any entry attempt in their respective sensor areas. The sensor gate in sensor area 1 is sensor 4 (S4), while the sensor 5 is the sensor gate in sensor area 2 (SA2). The sensor gate is the last sensor in the routing chain to transfer the data to the IDS. The selection of the sensor gate is not predetermined and depends on the transmission route whenever a transmission is to be engaged. In this scenario, sensor S1 starts transmission after the route has been established in a multi-hop communication manner. This means that the data is transferred from sensor to sensor until it reaches the sensor gate, which also relays it to IDS1. Once IDS1 receives the data, it will then encrypt the data using the public key (PK_2_) shared by the IDS2 during the initialization period. The encrypted message, E_M_, is now forwarded to IDS2 through γ_1_. Once IDS2 receives the data, it will go ahead and decrypt the message to be sent to sensor 5 (S5), which is the sensor gate in sensor area 2. The data or message is now transferred to the sink node or BS following the routes S6, S7, and BS, as viewed in Figure 10.

**Figure 11:**
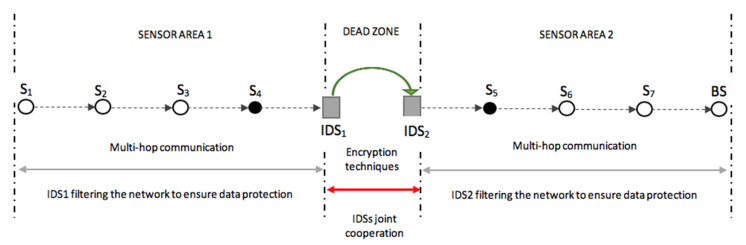
Schematic layout of the intercommunication between sensor areas with scaler IDS deployment.

Figure 11 shows the expanded schematic layout of the cooperation between IDSs in two sensor areas in a smart environment, as revealed in Figure 10. Figure 11 further expatiates the message transfer process (which is done by multi-hop communication techniques) from sensor area 1 (SA1) through IDS1 whiles it filters the data from its sensor area using a method based on the watchdog or sniffing method, and the functions of IDS1 and IDS2 by working together through encryption algorithms to overcome the dead zones between their respective sensor areas before the data is transmitted to the base station in sensor area 2 (SA2), with IDS2 also locally ensuring that the data received in its sensor area is authentic. 

**Figure 12:**
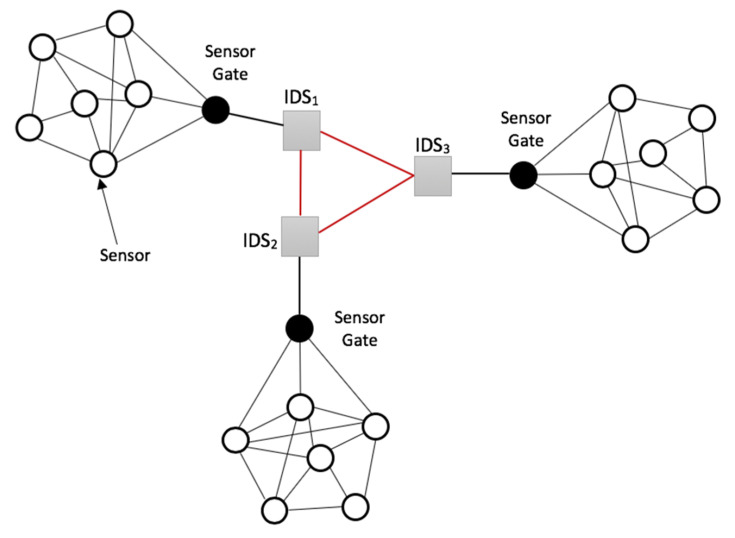
An overview of the IDS intercommunication hierarchy in a smart environment.

All IDSs present in a smart environment are equipped to intercommunicate to enable smooth and secure data processing and transmission in the whole region. This intercommunication is represented in Figure 12, showing an overview of a cluster hierarchy of communication between three (3) IDSs deployed in a smart environment. An IDS device does not only supervise the sensors in its sensor area but also affirms the security of the other IDSs in communication with it in the process of their joint cooperation mechanism through the novel developed cryptographic encryption algorithms.

### 3.4. Advantages of the Newly Proposed Intrusion Detection Device

The reliability of a security technique or method strongly depends on the conditions set for balanced and integrated security in general.

The advantages of the intrusion detection device compared to the other existing works are depicted in Table 1.

As indicated in Table 1, the reliability of a security technique or method strongly depends on the conditions set for general security. However, the list of these conditions has been expanded in this study to include other factors necessary to promote perfect security for both sensor networks and smart environments. Therefore, a proposed technique must be able to detect and isolate any intrusion, and it must provide a platform for the entire distributed network in a smart environment to be resilient to adapt, even in the event of an intrusion. By implementing all the computational functions, it must allow the sensors to benefit from a sufficient source of energy and memory capacity. Due to their diversity, the various works presented in the literature meet the different requirements in different ways, but not in a complete sense. When these techniques, in certain cases, meet safety conditions, they consume a lot of energy. The major problem is that some of the techniques only protect and/or guarantee the data that are forwarded between sensors. While some still manage to detect threats, their threat isolation remains problematic. Better still, the safety of smart environments remains a mystery for these proposed works, especially since they do not take into account the energy and the memory efficiency of the sensors. Some authors, such as Patil et al. [10], Singh et al. [11], Sonu et al. [12], Jamshidi et al. [13], Nadiammai and Hemalatha [43], and Almomani and Alenezi [42], presented techniques that address specific attacks. The downside to these techniques is that, in the event of different attacks, the entire network becomes extremely vulnerable. The immune system proposed by Patil et al. [10] is effective for the detection of danger signals capable of interfering with the signals transmitted in sensor networks. Still, such a system cannot preserve the integrity of all sensor networks that could be deployed over a large area (smart environment).

On the other hand, the novel intrusion detection device proposed in this study, by the concept of the virtual fence, ensures the integrity, the authenticity, the availability, the freshness, and the non-repudiation of the data because by sniffing the whole network and by filtering all the signals which are sent the outside network, the sensors can, therefore, ensure that all the messages received come from a reliable source. In other words, any sensor which receives a message or a route request knows that the latter has already been carefully checked by the detector (IDS) assigned to its zone. Therefore, all the messages transmitted are fresh and authentic. With this technique, the IDS solves the memory problem through the use of keys, as suggested by Zhang et al. [1], Gover et al. [2], Krontiris [3], Kumar et al. [4], Perrig et al. [5], Wang et al. [6], Zhu et al. [7], and Girao et al. [8]. In the same way, the IDS, by assuming all the computational functions of the whole network, allows the sensors to save their energy resources. Unlike the techniques presented in earlier existing works, the IDS allows total isolation of a threat in the event of detection. This detection and this isolation are only made possible by the introduction of the concept of the “trust table” and the feedback signal, which is a first in the field of security embedded in equipment other than the sensors, but acting as a sensor within the network. Finally, the table demonstrates that the IDS is capable of providing joint security for sensor networks and smart cities simultaneously, which is made possible by a spatial distribution of several IDSs in a smart city to prevent oversaturation of the computational functions of the IDSs and to ensure their effectiveness.

## 4. Conclusions

The rapid advancement of science over the past few decades in the field of telecommunications, and the pressing need for data collection, data processing, and data transmission, has led to new technology: wireless sensor networks (WSNs). A wireless sensor network, which is a set of interconnected sensors, is commonly used in several fields for data collecting, sharing, and forwarding. The rise of this new technology has brought a new touch in almost every domain. From weather forecasting, earthquake forecasting, mobile unit tracking, and agricultural forecasts, to border surveillance, WSNs tremendously aid humans in their daily lives. Examples include smart houses, smart parking, smart buildings, smart streets, and smart mobility, among others.

On the other hand, the sensitivity of the processed data or information by the sensors gives rise to attempts by malicious individuals who want to either intercept, damage, or corrupt this information. To provide better security schemes to WSNs, much work and research have been conducted. There are various existent methods that are widely accepted and used to provide security for wireless sensor networks. However, these works, which are mostly algorithms or cryptography-based in the sensors themselves, can affect the efficiency of the sensors and the entire network (in terms of energy consumption due to the micro sizes of the sensors).

In this paper, a different and novel approach has been proposed for providing a better security scheme for wireless sensor networks, especially in smart environments. The proposed security scheme, the intrusion detection system (IDS), is an electronic device with the characteristic of a sensor. This intrusion detection system computes the algorithms which are necessary for the detection and the isolation of any intruder or genuine node, which turns rogue in a WSN. The IDS generates a virtual fence around sensors by filtering all incoming and outgoing data transmission processes.

This isolation system is made possible by the implementation of a “trust table” (TT) system, which keeps records of all sensors in an IDSs’ sensor area used by the IDS to prove the authenticity of sensors and data transmission requests to prevent disruption of service. The isolation process of this novel system is further executed by the introduction of the Feedback Signal (F.S), an alert message broadcast about the malicious sensor, sent by the IDS to all genuine sensors in its sensor area to prevent future communication and message routing through the corrupted sensor.

The IDS also actively participates in the data transmission across sensor areas based on cooperation with other deployed or distributed IDSs over a large geographic environment by equipping the deployed IDS with the ability of data encryption and decryption. This is to eliminate the threats that may occur while the data is traveling from one sensor area to another through the dead zones, which are uncovered by the IDSs inside the smart environment.

Comparing this new intrusion detection system with the various existent mechanisms suggested for wireless sensor network security, the proposed IDS does not only tackle all the specific attacks related to WSNs but guarantees the entire network security. The IDS, compared to the existing works, releases the sensors from any computational functions, which might require more memory space and drain their energy. The IDS presents an all-in-one security solution for the WSNs by also resolving the security hole problem.

Future work would involve employing a new algorithm that enables the IDS to maintain a trust level with genuine mobile sensors (which do not belong to any sensor area), joining and leaving its communication range without being considered as malicious. This is in relation with the security mechanism of the IDS device where it supervises the wireless network to compare the number of sensors in its sensor area (where the sensors are mostly fixed in particular geographical positions) to maintain utmost security. With the advancement of technology in the future, mobile devices or sensors may be required to be employed in an already deployed region for particular purposes. The IDS therefore needs to recognize these mobile devices as being part of its sensor area and not as intruders.

## Figures and Tables

**Table 1:** The IDS advantages based on some security conditions.

Security Conditions	Proposed IDS	Proposed IDS Concept Ensuring the Conditions	Existing Works
Confidentiality	Yes	Virtual fence concept ensuring these conditions based on network traffic sniffing and F.S emission for isolation	Yes, but based on cryptography or some form of artificial intelligence
Authenticity	Yes		Yes, but based on cryptography or some form of artificial intelligence
Integrity	Yes		Yes, but based on cryptography or some form of artificial intelligence
Freshness	Yes		Yes, but based on cryptography or some form of artificial intelligence
Availability	Yes		Yes, but based on cryptography or some form of artificial intelligence
Non-repudiation	Yes		Yes, but based on cryptography or some form of artificial intelligence
Memory efficiency	Yes	IDS ensuring these conditions through computational function management	No
Energy Efficiency	Yes		No
Intrusion detection	Yes	Trust table and feedback signal concepts ensuring these conditions	Yes, but only largely for specific attacks
Intrusion isolation	Yes		No

## References

[1] Zhang X., He J., Wei Q. (2011). EDDK: Energy-efficient distributed deterministic key management for wireless sensor networks. Eur. J. Wirel. Commun. Netw..

[2] Grover J., Sharma S. Security issues in wireless sensor network—A review. Proceedings of the 2016 5th International Conference on Reliability, Infocom Technologies and Optimization (ICRITO) (Trends and Future Directions).

[3] Krontiris I. (2008). Intrusion Prevention and Detection in Wireless Sensor Networks. Ph.D. Thesis.

[4] Kumar Y., Munjal R., Kumar K. (2012). Wireless sensor networks and security challenges. Int. J. Comput. Appl. RTMC.

[5] Perrig A., Stankovic J., Wagner D. (2004). Security in wireless sensor networks. Commun. ACM.

[6] Wang Y., Attebury G. (2006). Byrav Ramamurthy A survey of security issues in wireless sensor networks. CSE J. Artic..

[7] Zhu S., Setia S., Jajodia S. (2006). LEAP: Efficient security mechanisms for large-scale distributed sensor networks. ACM Trans. Sens. Netw..

[8] Girao J., Westhoff D., Mykletun E., Araki T. (2006). TinyPEDS: Tiny persistent encrypted data storage in asynchronous wireless sensor networks. Ad Hoc Netw..

[9] Raj B., Mudali U.K. (2017). Sensor Science and Technology.

[10] Patil S., Chaudhari S. (2016). DoS attack prevention technique in wireless sensor networks. Procedia Comput. Sci..

[11] Singh R., Singh J., Singh R. (2016). WRHT: A hybrid technique for detection of Wormhole attack in wireless sensor networks. Mob. Inf. Syst..

[12] Sonu J.L., Malar K.J. (2017). Detection of Sybil Attack in Wireless Sensor Networks. Int. Res. J. Eng. Technol..

[13] Jamshidi M., Darwesh A.M., Lorenc A., Ranjbari M., Meybodi M.R. (2018). A precise algorithm for detecting malicious sybil nodes in mobile wireless sensor networks. IEIE Trans. Smart Process. Comput..

[14] Wazid M., Das A.K., Bhat K.V., Vasilakos A.V. (2019). LAM-CIoT: Lightweight authentication mechanism in cloud-based IoT environment. J. Netw. Comput. Appl..

[15] Challa S., Das A.K., Gope P., Kumar N., Wu F., Vasilakos A.V. (2020). Design and analysis of authenticated key agreement scheme in cloud-assisted cyber–physical systems. Futur. Gener. Comput. Syst..

[16] Saia R., Carta S., Recupero D.R., Fenu G. Internet of entities (IoE): A blockchain-based distributed paradigm for data exchange between wireless-based devices. Proceedings of the SENSORNETS 2019—Proceedings of the 8th International Conference on Sensor Networks.

[17] Alrajeh N.A., Khan S., Shams B. (2013). Intrusion detection systems in wireless sensor networks: A review. Int. J. Distrib. Sens. Netw..

[18] da Costa K.A.P., Papa J.P., Lisboa C.O., Munoz R., de Albuquerque V.H.C. (2019). Internet of Things: A survey on machine learning-based intrusion detection approaches. Comput. Netw..

[19] Godala S., Vaddella R.P.V. (2020). A study on intrusion detection system in wireless sensor networks. Int. J. Commun. Netw. Inf. Secur..

[20] Neisse R., Steri G., Fovino I.N., Baldini G. (2015). SecKit: A Model-based Security Toolkit for the Internet of Things. Comput. Secur..

[21] Kumari S., Rathi G., Attri P., Kumar M. (2014). Types of sensors and their applications. Int. J. Eng. Res. Dev..

[22] Pham C. (2013). Wireless Sensor Network: From Theory to Practice.

[23] Asim L. (2016). Anomaly Detection in Wireless Sensor Networks.

[24] Awodele O., Onuiri E.E., Okolie S.O. (2012). Vulnerabilities in Network Infrastructures and Prevention/Containment Measures. Proceedings of the Informing Science & IT Education Conference (InSITE).

[25] Sen J. (2009). A survey on wireless sensor network security. Int. J. Commun. Netw. Inf. Secur..

[26] Ekong V.E., Ekong U.O. (2016). A survey of security vulnerabilities in wireless sensor networks. Niger. J. Technol..

[27] Ouafaa I., Salah-ddine K., Jalal L., Said E.H. (2013). Review on the attacks and security protocols for wireless sensor networks. Eur. J. Sci. Res..

[28] Lupu T.-G. (2009). Main types of attacks in wireless sensor networks. Recent Adv. Signals Syst. 2.

[29] Messai M.-L. Classification of attacks in wireless sensor networks. Proceedings of the International Congress on Telecommunication and Application.

[30] Riaz M.N., Buriro A., Mahboob A. (2018). Classification of attacks on wireless sensor networks: A survey. Int. J. Wirel. Microw. Technol..

[31] Mpitziopoulos A., Gavalas D., Konstantopoulos C., Pantziou G. (2009). A survey on jamming attacks and countermeasures in WSNs. IEEE Commun. Surv. Tutor..

[32] Alquraishee A.G.A., Kar J. (2014). A survey on security in wireless sensor networks. Contemp. Eng. Sci..

[33] Santhi G., Sowmiya R. (2017). A survey on various attacks and countermeasures in wireless sensor networks. Int. J. Comput. Appl..

[34] Marigowda C.K., Shingadi M. (2013). Security vulnerability issues in wireless sensor networks: A short survey. Int. J. Adv. Res. Comput. Commun. Eng..

[35] Raymond D.R., Midkiff S.F. (2008). Denial-of-service in wireless sensor networks: Attacks and defenses. IEEE Pervasive Comput..

[36] Xing K., Srinivasan S.S.R., Rivera M., Li J., Cheng X., Huang S., MacCallum D., Du D.Z. (2005). Attacks and countermeasures in sensor networks: A survey. Network Security.

[37] Asha P.N., Mahalakshmi T., Archana S., Lingareddy S.C. (2016). Wireless sensor networks: A survey on security threats issues and challenges. Int. J. Comput. Sci. Mob. Comput..

[38] Kaplantzis S., Shilton A., Mani N., Şekerciǧlu Y.A. Detecting selective forwarding attacks in wireless sensor networks using support vector machines. Proceedings of the 2007 3rd International Conference on Intelligent Sensors, Sensor Networks and Information.

[39] Otoum S., Kantarci B., Mouftah H. Adaptively supervised and intrusion-aware data aggregation for wireless sensor clusters in critical infrastructures. Proceedings of the IEEE International Conference on Communications (ICC).

[40] Zhang R., Xiao X. (2019). Intrusion detection in wireless sensor networks with an improved NSA based on space division. J. Sens..

[41] Han L., Zhou M., Jia W., Dalil Z., Xu X. (2019). Intrusion detection model of wireless sensor networks based on game theory and an autoregressive model. Inf. Sci..

[42] Almomani I., Alenezi M. (2018). Efficient Denial of Service Attacks Detection in Wireless Sensor Networks. J. Inf. Sci. Eng..

[43] Nadiammai G.V., Hemalatha M. (2014). Effective approach toward Intrusion Detection System using data mining techniques. Egypt. Inform. J..

[44] Kamble J.R., Rangdale S.P. (2014). Intrusion detection using data mining techniques. Int. J. Sci. Res..

[45] Diro A.A., Chilamkurti N. (2018). Distributed attack detection scheme using deep learning approach for Internet of Things. Future Gener. Comput. Syst..

[46] Mansouri A., Majidi B., Shamisa A. (2018). Metaheuristic neural networks for anomaly recognition in industrial sensor networks with packet latency and jitter for smart infrastructures. Int. J. Comput. Appl..

[47] Wang H., Gu J., Wang S. (2017). An effective intrusion detection framework based on SVM with feature augmentation. Knowl. Based Syst..

[48] Koti M.R., Meshram M.D. (2013). An overview of advance microcontroller bus architecture relate on APB bridge. Int. J. Sci. Res. Publ..

[49] Biham E. (2005). Tutorial on Public Key Cryptography–RSA.

